# A Review of Modelling Test Study on the Effect of Single-Line Tunnelling on Adjacent Piles: Test Materials, Methodologies and Results

**DOI:** 10.3390/ma19112385

**Published:** 2026-06-03

**Authors:** Hongguo Diao, Yuhao Lu, Haibo Hu, Gang Wei, Qiang Li, Xiangyu Zhou

**Affiliations:** 1 School of Engineering, Hangzhou City University, Hangzhou 310015, China; diaohg@hzcu.edu.cn (H.D.); weig@hzcu.edu.cn (G.W.); 2 College of Civil Engineering and Architecture, Zhejiang University, Hangzhou 310058, China; 22512225@zju.edu.cn; 3 PowerChina Huadong Engineering (Shenzhen) Corporation Limited, Shenzhen 518100, China; qiangli1991@outlook.com; 4 China Railway No.3 Engineering Group Co., Ltd., Taiyuan 030001, China; zhouxiangyu945@163.com

**Keywords:** shield tunnel, existing piles, model test, test material, pile response

## Abstract

Tunnelling-induced safety risks from adjacent piles have become increasingly severe with the rapid development of urban underground space. Model tests have become essential for revealing the complex pile-tunnel interaction mechanism. This paper reviews the research progress of model tests on the influence of single-line tunnelling on adjacent piles, focusing on test soil materials, tunnel simulation methodologies, analysis of test results, and research prospects. However, current model test studies are constrained by several critical limitations, including insufficient similarity between soil materials and prototype conditions, and overly idealized simulation of tunnel excavation. This paper identifies a significant research gap: the inability of current volume-loss techniques to capture 3D dynamic factors (e.g., face pressure and grouting timing) and the lack of meso-scale observation at the pile-soil interface. This review provides a systematic synthesis of these methodological challenges and proposes future research prospects to provide a more scientific basis for engineering design and risk control.

## 1. Introduction

The situation of underground space construction has become increasingly severe with the acceleration of urbanization. When tunnels are excavated adjacent to existing piled foundations, the resulting ground movements would reduce the bearing capacity of the piles, leading to displacement of the supported structures while posing a safety hazard [1]. Different methods had been used to investigate the effects of tunnelling on adjacent piles. For theoretical and numerical analysis methods, it can be generally divided into global analysis method and two-stage analysis method. Numerical methods such as the finite element method (FEM) or finite difference method (FDM) were usually employed in global analysis method, which treated the pile and the surrounding soil as an integrated system during excavation simulation, allowing consideration of pile-soil interaction, pile-pile interaction in group, soil anisotropy, and complex boundary conditions [2,3,4,5]. Furthermore, fully coupled three-dimensional numerical analyses have been extensively adopted to establish the baseline for the global interaction mechanism between single-line tunnels and adjacent pile groups [6,7]. The two-stage analysis method divided the analysis into two steps, where the free field soil displacements induced only by tunnelling without existing piles were firstly calculated, and then the displacement results were applied to the piles using the boundary element method or load transfer method to analyze the additional deformation and internal force of the pile [8,9,10,11].To improve computational efficiency, advanced boundary-element and simplified elastic solutions have also been introduced into the two-stage analysis framework, providing robust predictions for pile deformation [12,13,14].

For field measurement method, the earliest report could be traced back to 1986 when Attewell et al. [15] studied a tunnel passing sideways through an existing pile-supported structure and found that the tunnelling-induced negative shaft friction on the piles could be reduced by applying an asphalt coating to the pile body. Subsequently, an engineering case in London was reported where a tunnel passed sideways through adjacent pile-supported buildings with a minimum horizontal clearance of about 1 m in London stiff clay, and a maximum horizontal pile displacement of up to 10 mm was observed subjected to tunnelling with a volume loss at 2% [16,17]. Another construction case of an open shield tunnel passing sideways through existing pile-supported structures on the Island Line of the Hong Kong MTR was reported, and a maximum pile settlement of 12 mm (0.6% of the pile diameter) was measured [18]. The additional pile responses induced by tunnelling were further found to be related to the relative position of the tunnel and the pile, including the transverse clearance and the longitudinal relative distance between the tunnel face and the pile [19,20,21,22]. Regarding the construction case of tunnelling beneath the existing piles, Khoo et al. [23] reported a full-scale study in Malaysia and found that negative shaft friction was observed in pile with a maximum downdrag load reaching between 56% and 71% of the piles working load. Ma. et al. [24] also observed that negative shaft friction was developed below the neutral points in the pile and the pile bearing capacity was reduced. In General, field reports were still limited as the existing piles were deeply buried underground, resulting in difficulty in installing the sensors. Furthermore, current measured data predominantly included pile displacements, surrounding soil and superstructure, whereas pile axial force and bending moment were observed only in isolated cases. In complex urban environments, the kinematic interaction is further complicated by the stiffness of the superstructure, necessitating analyses that consider the entire piled raft foundation system subjected to single-line tunnelling movements [25].

**Figure 1:**
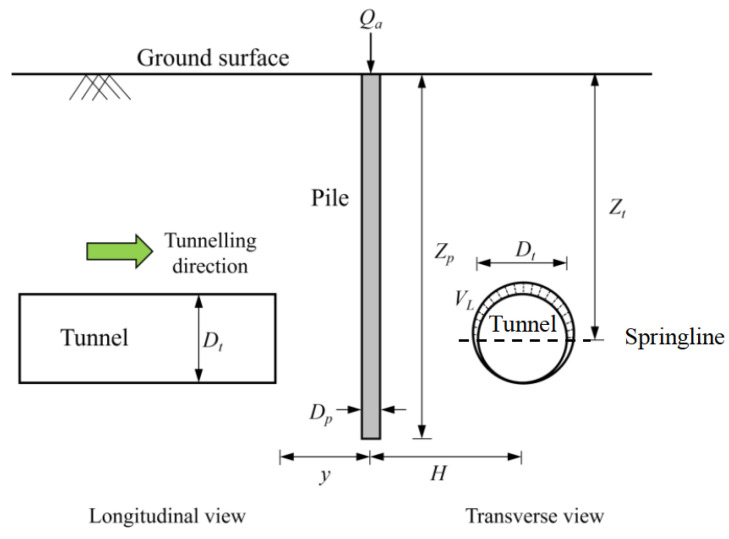
Relevant parameters in pile-tunnel interaction problem. Note: $C_{u}$ is the non-uniformity coefficient, defined as $C_{u}=D_{60}/D_{10}$, where $D_{60}$ and $D_{10}$ represent the particle sizes corresponding to 60% and 10% finer by weight, respectively.

Compared with field measurement method, model test method offered advantages of lower cost, easier operation, the ability to capture data difficult to measure in field, and the capacity for parametric analyses. Key parameters influencing pile-tunnel interaction in model tests are illustrated in Figure 1, where $D_{t}$ is the tunnel diameter, $V_{L}$ is the tunnel volume loss, $Z_{t}$ is the tunnel depth, $D_{p}$ is the pile diameter, $Z_{p}$ is the pile depth, $Q_{a}$ is the pile working load (or load factor LF, defined as the ratio of pile working load to ultimate capacity), y is the longitudinal distance from the tunnel face to the pile centerline, and H is the transverse horizontal distance between the centerline of tunnel and pile. These parameters are commonly varied to investigate the effects of single-line tunnelling on adjacent piles. While existing reviews often focus broadly on field measurements or numerical methods, a systematic synthesis specifically isolating the scaling effects, soil simulation limits, and mechanical tunnel-simulation methodologies in physical modeling has been lacking.

Specifically, while field measurements provide indispensable macro-data, they are inherently limited by the extreme difficulty of sub-surface sensor installation and the inability to isolate individual influence factors in complex urban environments. Numerical methods, although computationally efficient, rely heavily on constitutive models that struggle to capture the highly non-linear and discontinuous kinematic interactions at the pile-soil-tunnel interface. Physical model testing (both 1 g and n g) offers a unique middle ground to resolve these issues; however, the current body of research is critically fragmented. This review identifies a major research gap: existing physical modeling studies often overlook the profound discrepancies between idealized laboratory conditions and real-world complexity. This includes the failure of conventional volume-loss techniques to replicate 3D dynamic factors (such as face pressure and grouting timing) and the insufficient scaling similarity of soil materials. By systematically isolating these technical bottlenecks—soil similarity, simulation realism, and observation precision—this paper justifies the urgent need for a methodological framework that transitions from descriptive cataloging to critical experimental design. Model test studies on the influence of single-line tunnelling on adjacent piles were reviewed in this paper. Various test soil materials, tunnel simulation methodologies and test results were summarized and compared, and research prospects were proposed to guide subsequent studies.

## 2. Test Soil Materials

Granular materials are commonly adopted as the soil in geotechnical tests. However, most of the experiments used sandy soil or clayey soil due to the complexity of the granular materials [26,27,28,29]. The physical and mechanical property index of sandy soil and clayey soil in reported model tests are summarized and listed in Table 1 and Table 2, respectively. In the normal gravity scaled model test, particle size effects would be negligible when the soil particle size was less than 1/30 of the characteristic model dimensions [30]. In the hypergravity centrifuge model test, it was proposed that particle size effects diminished when the ratio of tunnel diameter to mean particle size exceeded 350 and could be ignored when the ratio exceeded 500 [31,32]. Since the particle sizes used in the reviewed tests are all smaller than these thresholds (i.e., less than 1/30 of the model dimensions for 1 g tests, or a tunnel diameter to mean particle size ratio exceeding 500 for centrifuge tests), particle size effects can be considered negligible.

**Table 1:** Physical and mechanical property index of sandy soil in reported model tests.

Reference	Material	Specific Gravity *G*S	Average Particle Size (mm)	Non-Uniformity Coefficient *C*u	Relative Density *Dr*	Angle of Internal Friction *φ* (°)	Void Ratio *e*	
							*e*max	*e*min
Ghahremannejad et al. [33]	Sydney dry sand	2.65	0.30	1.47	79–87%	-	0.880	0.656
Jacobsz et al. [34]	Dry silica sand	2.67	0.09–0.15	1.60	75% ± 2%	-	-	-
Lee and Chiang [35]	Quartz sand	2.65	0.18	1.58	65% ± 2%	34	-	-
Marshall and Mair [36]	Grade E dry silica sand	2.67	0.12	-	90%	-	0.970	0.640
Boonsiri and Takemura [37]	Toyoura dry sand	2.65	0.19	1.62	80%	40	0.973	0.609
Franza and Marshall [38]	Dry silica sand	-	0.12	-	30%	-	-	-

As shown in Table 1, Sydney sand, silica sand, quartz sand and Toyoura sand without considering the influence of pore water were commonly used as sandy soil, with the specific gravity around 2.65, average particle size of 0.09~0.30 mm, non-uniformity coefficient of 1.47~1.62, and relative density generally greater than 65% (i.e., dense sand). The determination of the maximum and minimum dry densities for calculating relative density follows the standards established in ASTM D4253 and ASTM D4254 [39,40]. The sand raining method was commonly adopted for specimen preparation [34], where sand was pluviated from a constant vertical distance to achieve desired density and uniformity.

**Table 2:** Physical and mechanical property index of clayey soil in reported model tests.

Reference	Material	Consolidation State	Bulk Density (kN/$m^{3}$)	Moisture Content (%)	Shear Strength (kPa)	Coefficient of Consolidation ($m^{2}$/year)
Meguid and Mattar [41]	Bentonite mixture	Normal consolidation	17.9	40	3~4	-
Loganathan et al. [42]	Kaolin	overcosolidation	16.5	120	75	2
Williamson et al. [43,44]	Speswhite kaolin	overcosolidation	17.5	-	50~80	0.5–1.2
Ong et al. [45,46]	Malaysian kaolin	overcosolidation	-	120	15	-
Hartono et al. [47]	Kaolin	Normal consolidation	16.0	120	3 + 1.8*z* (*z* is soil depth, unit: m)	20
McNamara et al. [48]	Speswhite kaolin	overcosolidation	17.5	-	-	-
Ma et al. [49,50]	Expansive clay in Guangxi, China	overcosolidation	19.0	18	-	-
Gu [51]	Silty clay in the floodplain area of Yangtze River in Nanjing, China	Normal consolidation	18.2	33.6	1.65*z* (*z* is soil depth, unit: m)	-

As shown in Table 2, various types of bentonite mixtures or kaolin were commonly used as clayey soil, with different soil sample preparation methods. A mixture composed of 44% fine sand, 10% bentonite, 4% cement, and 42% water was used by Meguid and Mattar [41]. Kaolin mixed to a slurry with a nominal water content of 120% (twice the liquid limit) in a required ultimate consolidation stress state was adopted by Loganathan et al. [42]. Both a stiff clay and softer clay with a preconsolidation pressure of 800 kPa and 400 kPa, a shear strength of 80 kPa and 50 kPa respectively were used by Williamson et al. [43,44]. Some special soils such as the expansive clay from Guangxi in China, with a maximum dry density of 1.88 g/cm^3^, plasticity index of 28.0, optimum moisture content of 12.9%, and free swelling ratio of 59%, were also investigated in model test [49,50].

**Figure 2:**
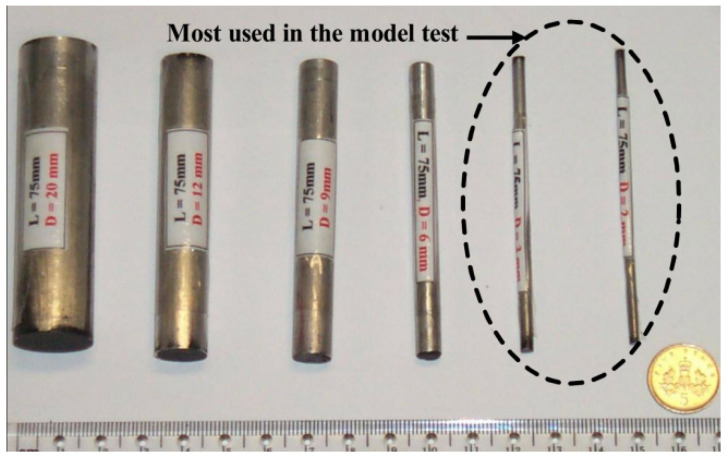
Aluminum rod model [52].

In addition to the aforementioned sandy and clayey soils, other materials like special granular material and transparent soil had also been used in model tests. A mixture of aluminum rods of various sizes in Figure 2 was used to represent a well-graded two-dimensional granular material [52,53]. Medium-dense crushed glass with a particle size of 2~3 mm and internal friction angle of 39° was used behaving similar to angular silica sand [54]. Transparent soil had been used in geotechnical tests as its geomechanical properties was similar to natural soils and the internal deformation could be visualized combined with particle image velocimetry technology [55]. Transparent soil made from fused quartz sand and a 4:1 mixture of 15# white oil and n-dodecane (CH_3_(CH_2_)_10_CH_3_) was used to study the effect of tunnelling-induced ground volume loss on adjacent piles [56]. A similar transparent soil was also adopted to investigate the pile responses subjected to a DOT shield tunnel excavation [57].

## 3. Tunnel Simulation Methodology

Due to the complexity of real tunnel excavation processes, it is difficult to fully reproduce each construction step in laboratory model tests. The ground deformation caused by tunnelling is usually related to multiple factors, including tunnel face condition, shield machine advancing, tail voids, lining deformation, soil reconsolidation, etc. These factors are collectively represented by the tunnel volume loss ($V_{L}$), which is defined as the ratio of the additional volume of ground excavated to the theoretical volume of the finished tunnel cross-section. The common tunnel simulation methodologies in normal gravity scaled model tests (1 g) and hypergravity centrifuge model tests (n g) are presented in Table 3. It revealed that the tunnel volume loss had been the most widely adopted control parameter for simulating tunnel excavation in such tests.

**Table 3:** General tunnel simulation methodology in reported model tests.

Test Type	Reference	Tunnel Simulation Method	Tunnel Simulation Methodology
Normal gravity scaled model test (1 g)	Morton and King [58], He et al. [59], Guo et al. [60]	Shrinkage method	The tunnel excavation was simulated by rotating to excavate the internal soil in the hollow circular pipe. (Figure 3a,m,s)
	Ghahremannejad et al. [33]	Shrinkage method	Four hollow aluminum tubes with different diameters and nested in sequence could simulate a $V_{L}$ of 2%, 4%, and 6% respectively through sequential telescoping. (Figure 3b)
	Lee and Yoo [52,53] Lee and Bassett [61]	Shrinkage method	A cylinder composed of six connected segments was regarded as the tunnel lining and the gasket inside could be radially shrunk by rotation to simulate a $V_{L}$ between 4% and 20%. (Figure 3c,p)
	Broere and Dijkstra [54]	Shrinkage method	Two semi-circular steel pipes was composed as the tunnel lining and a $V_{L}$ of 0.6% was simulated by longitudinal contraction through a built-in stepper motor. (Figure 3d)
	Shahin et al. [62,63]	Shrinkage method	12 peripheral segments through the central axis were controlled to simulate two tunnel displacement modes, namely uniform radial displacements and non-uniform radial displacements with $V_{L}$ between 0.2% and 15.36%. (Figure 3f,o)
	Liu et al. [64]	Shrinkage method	A shield machine model was consisted of shell, cutterhead, traveling guide rail, shield excavator, organ cover and hydraulic station, which the cutterhead could rotate and move forward to cut the soil through the hydraulic station to simulate the tunnelling. (Figure 3t)
Hypergravity centrifuge model test (n g)	Hergarden et al. [65], Bezuijen and van der Schrier [66]	Shrinkage method	A ring structure formed by four sections connected in a zigzag pattern to simulate a $V_{L}$ up to 8% through the radial contraction of the core structure. (Figure 3g,q)
	Loganathan et al. [42]	Drainage method	A certain volume of oil was injected into the gap between the inner rigid tube and the outer annular rubber membrane, and then different $V_{L}$ could be achieved by discharging the oil in uniform radial displacements mode. (Figure 3h)
	Ran [67]	Dissolution method	The interior of a copper foil-made tunnel lining was filled with high-density polystyrene foam and a $V_{L}$ of 4.2% could be simulated by injecting an acetone solution to dissolve the foam by stages. (Figure 3e)
	Ong et al. [45]	Dissolution method	Foam was filled in the rubber membrane inside the rigid lining and a $V_{L}$ of 3.3% in non-uniform radial displacements mode was achieved by injecting a solvent to dissolve all foam at once. (Figure 3i)
	Hartono et al. [47]	Drainage method	Water was filled in the gap between the rigid circular tube and the outer annular rubber membrane and then different $V_{L}$ could be simulated by drainage in non-uniform radial displacements mode. (Figure 3j)
	Jacobsz et al. [34]	Drainage method	A certain volume of water was injected into the gap between the inner copper tube and the outer annular rubber membrane, and then a $V_{L}$ ranging from 0% to 20% under plane-strain condition was achieved by discharging all water at once. (Figure 3h)
	Lee and Chiang [35]	Airbag method	The main structure was a cylindrical airbag reinforced with fiber tape, and tunnel excavation was simulated by reducing the air pressure. (Figure 3e)
	Marshall [32]	Drainage method	The gap between rigid brass cylinder and exterior flexible rubber membrane was filled with water and tunnelling was achieved by drainage. (Figure 3j,n)
	Wu et al. [68], Su et al. [69]	Drainage method	A multi-section cylindrical rubber bag filled with heavy liquid was used as the tunnel model and tunnelling was simulated by the pressure balance and the discharge of heavy liquid. (Figure 3k,r)
	Boonsiri and Takemura [37]	Shrinkage method	A steel ring supported by a retractable conical gasket and covered with two layers of outer rubber films was adopted to simulating the tunnelling with a maximum $V_{L}$ of 15%. (Figure 3l)
	Williamson et al. [43,44]	Drainage method	A rigid copper tube and an exterior flexible rubber membrane with water filling the gap were adopted to simulating the tunnelling. (Figure 3j)
	Song and Marshall [70,71]	Shrinkage method	$V_{L}$ ranging from 0.11% to 3.5% was achieved by rotating the bi-directional screw shaft, which caused two hexagonal wedge-shaped shafts to move in opposite directions, causing the relative movement between the six individual segments according to the different taper angles of the wedge-shaped shafts. (Figure 3u,v)

**Figure 3:**
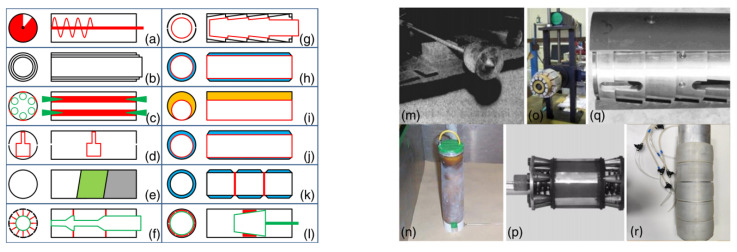

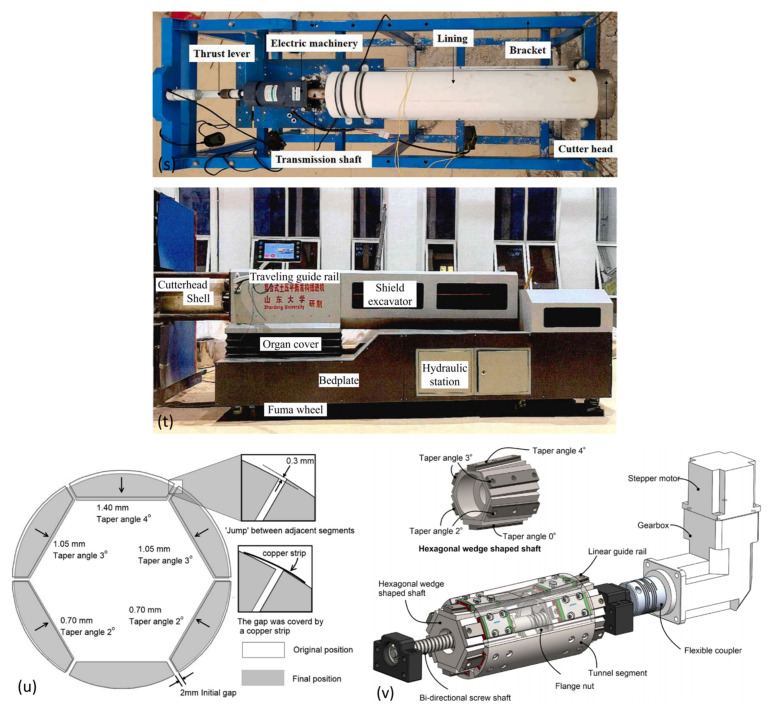
Schemes and photos of different tunnel simulation methodologies: (**a**–**q**) were modified from Dias and Bezuijen [72]; (**r**) was reprinted from Wu et al. [68]; (**s**) was reprinted from He et al. [59]; (**t**) reprinted from Liu et al. [64]; (**u**,**v**) were reprinted from Song and Marshall [70].

In summary, current tunnel simulation methodologies can be broadly categorized into four primary types: shrinkage, dissolution, airbag, and drainage methods. These approaches exhibit distinct characteristics in terms of realism and applicability across different testing scales. Normal gravity (1 g) tests frequently utilize the mechanical shrinkage method, which can incorporate complex shield models to better replicate the three-dimensional mechanical excavation process, though they are limited in simulating the prototype soil stress field. In contrast, hypergravity centrifuge (n g) tests achieve higher stress-field fidelity but predominantly rely on simplified volume-loss techniques, such as drainage, dissolution, or airbag methods. While these fluid, foam, or air-based methods effectively control the global volume loss, they often overidealize the non-uniform ground movement and lack the mechanical realism of actual shield cutting. Recognizing these methodological advantages and limitations is essential for critically interpreting the diverse pile-tunnel interaction mechanisms observed across different studies.

## 4. Analysis of Test Results

Current model tests of single-line tunnel primarily investigated the effects of pile-tunnel relative position, tunnel volume loss, pile working load, and pile type on pile responses. Compared with the ground surface settlements subjected to tunnelling investigated by Jacobsz et al. [34], Kaalberg et al. [73] and Selemetas [74], the zone of influence around the tunnel in which the potential for larger pile settlements exists is summarized. Existing studies have summarized the tunnel influence zone associated with larger pile settlements, which is a sector-shaped region extending outward from the tunnel and differs from the surface settlement influence range. The boundary angle of this influence zone is generally defined by a range of 45° to 60°, and its scope is jointly controlled by the tunnel diameter, soil properties and volume loss [4]. Overall two mechanisms have been described for the tunnel effects on piles: (1) The tunnel degrades the pile base capacity, which requires a mobilization of shaft friction with limited settlements, and once the shaft is fully mobilized, higher settlements are necessary to recompress and mobilize the base. (2) The base capacity is not degraded and the relative pile-soil settlements induce negative friction on the pile shaft, which increases the base load [72]. In the following analysis of test results, the tests are defined as two categories, normal gravity scaled model test (1 g) and hypergravity centrifuge model test (n g). It should be emphasized that the diverse pile-tunnel interaction mechanisms and ‘zones of influence’ reported across the various studies reviewed in this section are inherently governed by the specific scaling laws, boundary constraints, and soil preparation methods detailed in Section 2 and Section 3. These methodological factors are crucial for a critical and comparative interpretation of the experimental findings presented below.

### 4.1. Normal Gravity Scaled Model Test (1 g)

The earliest scaled model test for the pile-tunnel interaction problem could date back to 1979, when Morton and King [58] indicated that pile bearing capacity and settlement were significantly affected by tunnelling in soft soil. Ghahremannejad et al. [33] found that the maximum pile bending moment was measured about 1.5$D_{t}$ above the springline (i.e., the horizontal centerline and widest point of the tunnel cross-section) and increased with volume loss during single-line tunnelling near the pile toe in dense sand. Meguid and Mattar [41] investigated the interaction between single tunnel and pile group in clay, indicating that the bending moment of tunnel lining decreased with the reduction in pile-tunnel distance while the distance was less than 1$D_{t}$.

Two-dimensional model tests were conducted using aluminum rods and the close-range photogrammetric technique, showing that pile displacement was strongly influenced by the pile-tunnel relative position [52,53,61]. An influence zone for pile settlement was also proposed, which was dominated by pile tip location, pile working load, pile size, tunnel volume loss, soil strength, granular material dilatancy and tunnel size. Crushed glass was applied as a photoelastic material to analyze the effects of volume loss on soil stress and adjacent pile, implying that a significant change in the tip stress in displacement piles was exhibited with a volume loss of 0.6% [54]. Extensive physical modelling indicates that the maximum lateral deflection of piles strongly correlates with the horizontal distance to the advancing tunnel face [75]. The horizontal deformation mechanism of adjacent pile under different pile diameters, tunnel diameters, tunnel volume loss and different position conditions was revealed when considering both radial and longitudinal influences along the tunnel in silty clay from the Nanjing Yangtze River floodplain [51]. Significant additional internal forces and displacements of the pile group (2 × 2) were induced by tunnelling near the pile toe in clay. However, these responses decreased quickly with increasing horizontal pile-tunnel distance. Furthermore, the bearing capacity of the piles was weakened due to the development of negative shaft friction [59]. Several model tests were carried out to study the effects of tunnelling on unilateral and bilateral pile group (1 × 4) in sandy pebble stratum, proposing that the vertical and horizontal displacements of the pile group would be affected by pile lengths and pile-tunnel distance due to the sheltering effect in pile group [58]. This sheltering effect and the overall pile group settlement are highly dependent on the pile group stiffness and the configuration size, which dictate the distribution of the tunneling-induced forces [76].

### 4.2. Hypergravity Centrifuge Model Test (n g)

Hypergravity centrifugal model tests have been increasingly adopted to study the pile-tunnel interaction problem, as they can reproduce the actual stress field at the model scale. Recent advancements in hypergravity centrifuge testing have successfully isolated the complex pile load-transfer mechanisms and soil stress relaxations induced by single-line tunnelling in uniform sand [77]. Most such tests have been conducted in homogeneous sandy soil or clayey soil, as listed in Table 4.

**Table 4:** Reported centrifugal model tests.

Reference	Soil Material	Model Pile	Test Procedure
Jacobsz et al. [34]	Grade E dry silica sand	Single pile: aluminum alloy pile with a free pile head and working load (LF = 0.5).	Model pile was pre-set first at 1 g and then a pneumatic device was used to drive the pile to the designed depth at 75 g. After stabilization, the water was gradually discharged to simulate the tunnelling.
Feng et al. [78]	Toyoura dry sand	Single pile: aluminum alloy hollow square pile with a free pile head and zero working load.	Model pile was installed at 1 g and the tunnel passing sideways through the pile was simulated by dissolving the foam after stabilization at 100 g.
Lee and Chiang [35], Lee et al. [79]	Quartz sand	Single pile: aluminum alloy hollow circular tube with a free pile head and working load (LF = 0, 0.25, 0.5).	Model pile was installed before sand spreading at 1 g and then the pressure of the airbag was gradually reduced to simulate the tunnelling after the stabilization process at 100 g.
Marshall and Mair [36]	Grade E dry silica sand	Single pile: aluminum alloy semi-cylinder with a free pile head and working load (LF = 0.625).	A half-mold model pile was installed closely against the transparent plexiglass during the sand pouring process at 1 g and fully penetrated to the designed depth at 75 g, following the tunnel simulation.
Boonsiri and Takemura [37]	Toyoura dry sand	Pile group (2 × 2): acrylic pipes with a pile cap and working load (LF = 0.4, 0.67).	Pile group were installed before sand pouring at 1 g, and then the tunnel passing sideways through the pile group on the side was simulated at 100 g.
Franza and Marshall [38]	Silica dry sand	Single pile: aluminum alloy circular pile with a free pile head and working load (LF = 0.67).	Complete non-displacement pile and part of displacement pile was jacked prior to spin up at 1 g. After stabilizing at 60 g, the working load was applied directly for non-displacement pile, or the remaining pile section was jacked in-flight and then working load was applied for displacement pile, following the tunnel simulation at last.
Loganathan et al. [42]	Kaolin	Single pile & pile group (2 × 2): copper tube with a working load (LF = 0.5).	Model tunnel and piles were placed at 1 g after completing the soil consolidation at 100 g, and, subsequently, the tunnelling under plane-strain condition was simulated after pore pressure was dissipated at 100 g again.
Williamson et al. [43,44]	Homogeneous stiff clay and soft clay	Single pile: rectangular aluminum alloy tube with a free pile head and working load (LF = 0.3~0.5).	Model tunnel and pile were placed at 1 g. After soil consolidation at 75 g, the working load was applied and then the tunnelling beneath the pile was simulated.
Ong et al. [45,46]	Malaysian kaolin	Single pile: aluminum alloy hollow square pile, with a free pile head and no load on the pile top.	Model pile was installed at 1 g and the tunnel passing sideways through the pile was simulated by dissolving the foam after soil consolidation at 100 g.
Hartono et al. [47]	Overconsolidated kaolin	Single pile: aluminum alloy circular tube with a free pile head and zero working load.	Model pile was installed at 1 g and the tunnelling beneath the pile was simulated after soil consolidation at 100 g.
McNamara et al. [48]	Kaolin	Pile group (2 × 2): teat-treated steel pipes with a pile cap.	Pile group was installed at 1 g and the tunnelling was simulated by gradually depressurizing the airbag after soil consolidation at 100 g.
Ma et al. [49,50]	Expansive clay in Guangxi, China	Pile group (2 × 2): aluminum pipes with a pile cap and working load (LF = 0.4).	Model tunnel and piles were placed at 1 g, and the working load was applied after soil consolidation at 40 g, following the tunnel simulation.

In sandy soils, the influence zone of tunnelling on adjacent pile was first classified into four types based on the relationship between pile settlement and ground surface settlement. This classification divides the space around the tunnel into distinct regions with reference to a boundary angle of 45° plus half the internal friction angle of the soil and distinguishes zones with a pile settlement greater than 20 mm and less than 20 mm to quantify the tunnelling impact level [34]. The response of an adjacent single pile without working load to tunnelling was studied, and it was revealed that the maximum pile axial force and bending moment exhibited at the tunnel springline depth [78]. Ground surface settlement induced by tunnelling and its impact on adjacent single piles were examined, and it was found that pile bending moment was primarily governed by the vertical pile-tunnel relative position, while pile axial force was determined by both vertical pile-tunnel relative position and pile working load [35,79]. The influence of tunnelling on pile bending moment and axial force was studied by installing end-bearing piles within different influence zones, and the relationship between pile-tunnel relative position and soil loss at the onset of significant pile settlement was established [36]. The impact of tunnelling on adjacent pile group was further studied through a series of centrifugal model tests based on the influence zone division [37]. Tunnelling-induced pile failure was defined as occurring when ultimate bearing capacity was reduced below the pile working load [38]. Furthermore, centrifuge tests explicitly incorporating superstructure stiffness have demonstrated that the presence of a rigid frame significantly redistributes the tunnelling-induced settlements and mitigates severe differential pile movements [80]. Their findings indicated that tunnel excavation was more likely to cause failure in displaced piles with a lower safety factor, and a larger tunnel volume loss corresponding to failure in non-displacement piles was needed than that in displacement piles with an equivalent safety factor.

In clayey soils, the behaviors of single pile and pile group subjected to tunnelling in varying depth were investigated, revealing that the maximum additional pile bending moment exhibited an approximately linear relationship with tunnel volume loss [42]. Clay consolidation state and model box temperature were further taken into account to reveal the pile responses caused by tunnelling sideways and beneath the pile [43,44]. Short-term and long-term additional responses of a single pile induced by adjacent tunnelling were studied, finding that long-term pile response was significantly influenced by pore pressure dissipation and soil consolidation degree, and the short-term surface settlement trough generally followed a Gaussian distribution, whereas the long-term trough assumed a parabolic shape [45,46]. Negative shaft friction was observed developing in the upper pile portion due to tunnelling beneath the pile when the horizontal distance between pile and tunnel axis reached 2$D_{t}$ [47]. Horizontal soil displacement around the tunnel was found to be amplified, with the surface settlement trough shifting toward the pile side due to the presence of the pile during tunnelling [48]. Regarding regional expansive clay, the long-term effects of tunnelling on pile group were also investigated, revealing that piles continued settling even after the tunnel had passed the pile due to the pronounced three-dimensional and time-dependent characteristics of tunnelling. Beyond that, the pile responses to cyclic water level fluctuations were also analyzed by considering the wetting-drying property of expansive clay [49,50].

## 5. Research Prospect

Although model tests have yielded valuable insights into the mechanism of pile-tunnel interaction, several important issues in current research require further exploration and resolution, as outlined in the following.

(1) The similarity between the soil material and the prototype conditions is insufficient. Homogeneous dry sand or remolded kaolin were commonly applied in most studies, which differ significantly from natural undisturbed soils in stress history, structure, and anisotropy [81,82,83]. Time-dependent characteristics (e.g., creep, consolidation) in soft clay are difficult to simulate fully, and groundwater seepage is rarely considered especially. Moreover, research on tunnelling in complex layered strata (e.g., soft-over-hard strata, interbedded strata) and unsaturated soils is lacking. Therefore, a concrete research priority is to develop advanced testing apparatuses capable of incorporating groundwater seepage and simulating complex interbedded strata.
(2) The simulation of the tunnelling process is overly idealized. Existing methods mainly simulated uniform radial displacements under two-dimensional plane strain conditions. However, actual shield tunnelling is a three-dimensional dynamic process involving coupled factors (e.g., tunnel face pressure, shield friction, grouting pressure and timing) that produce a non-uniform three-dimensional displacement field, which current models cannot realistically replicate. Future studies should prioritize developing novel mechanical shield models that can decouple these 3D dynamic factors to replace conventional volume-loss techniques.
(3) The means of observation and the depth of mechanism revelation are limited. Most tests relied on external sensors for macroscopic measurements, failing to capture meso-scale behavior at the pile-soil interface, the evolution of soil arching around the tunnel, or pile-soil-pile interaction in group. There is a lack of full-field, non-contact, high-precision observations. Visualization techniques like transparent soil are still in their infancy, mainly applied to sands, and suffer from issues such as transparency degradation, drying, and hardening during testing. Overcoming these specific material degradation issues in transparent soils represents an urgent technical direction.
(4) Research on long-term effects and cyclic loading influences is insufficient. Apart from a few studies on the soil consolidation in clay and wetting-drying property in expansive soil, systematic model tests on cumulative effects of long-term phenomena (e.g., post-construction creep, water level fluctuations, traffic loads) on pile performance are lacking. The transition from short-term to long-term safety after construction needs to be considered.
(5) Prediction methods and design guidelines with general applicability are lacking. Most experimental findings were summaries of patterns under specific working conditions, and although parametric studies were numerous, the conclusions were rather fragmented. While it is well recognized that pile-tunnel relative position, tunnel volume loss, and pile working load are the key factors, integrating these complex mechanisms into simplified analysis models or design charts that account for multi-factor coupling remains a challenge.
(6) In addition, in recent years, a series of research methods and experimental approaches that can be referenced have emerged in the areas of underground structure stress analysis, mesoscopic observation of soil, regional geotechnical material characteristics, and mechanical behavior of composite structures. These include the analysis of chloride ion transport in submarine tunnels [84], characterization of the mesoscopic shear mechanism of two-dimensional granular materials [85], study of asymmetric foundation pit spatial effects [86], atmospheric erosion durability testing of artificial sand concrete [87], investigation of pile foundation concrete deterioration under the coupling of stray current and chloride ions [88], mechanical property tests of reinforced silty soil from the Qiantang River [89], analysis of the stress mechanism of steel–concrete composite connections [90], and nonlinear numerical simulation methods for prefabricated steel–concrete piers [91]. The aforementioned research methods and technical approaches can provide important references for the design optimization, enhancement of observation techniques, and in-depth mechanistic studies of subsequent tunnel–pile interaction model experiments.

## 6. Conclusions

Although tunnelling generally does not cause catastrophic damage in adjacent existing piles, its effects on pile displacements cannot be ignored and must be controlled within acceptable limits. Hence, studying the influence of tunnelling on adjacent piles remains practically important. Model tests are indispensable for revealing the complex pile-tunnel interaction mechanism, which compensate for the scarcity of field-measured data through controllable conditions and offer unique advantages for parametric sensitivity analysis and mechanism exploration. Soil materials, tunnel simulation methodologies, and analysis of test results in physical model tests were reviewed, as well as deficiencies and future research prospects in this field were identified in this paper.

## Data Availability

The original contributions presented in this study are included in the article. Further inquiries can be directed to the corresponding author.

## References

[1] Sohaei H., Namazi E., Hajihassani M., Marto A. (2020). A review on tunnel-pile interaction applied by physical modeling. Geotech. Geol. Eng..

[2] Lee G.T.K., Ng C.W.W. (2005). Effects of advancing open face tunneling on an existing loaded pile. J. Geotech. Geoenviron. Eng..

[3] Poulos H.G. (2011). Comparisons between measured computed responses of piles adjacent to tunnelling operations. Geotech. Lett..

[4] Zheng G., Wang R., Lei H., Zhang T., Fan Q. (2023). Load-transfer-associated settlements of a piled building during shield tunneling in soft ground. Tunn. Undergr. Space Technol..

[5] Soomro M.A., Liu K., Cui Z.D., Mangi N., Mangnejo D.A. (2024). Insights from 3D numerical simulations on the impact of tunnelling on vertical battered pile groups under lateral loading. Comput. Geotech..

[6] Mroueh H., Shahrour I. (2002). Three-dimensional finite element analysis of the interaction between tunneling and pile foundations. Int. J. Numer. Anal. Meth. Geomech..

[7] Soomro M.A., Mangnejo D.A., Bhanbhro R., Memon N.A., Memon M.A. (2019). 3D finite element analysis of pile responses to adjacent excavation in soft clay: Effects of different excavation depths systems relative to a floating pile. Tunn. Undergr. Space Technol..

[8] Huang M., Zhang C., Li Z. (2009). A simplified analysis method for the influence of tunneling on grouped piles. Tunn. Undergr. Space Technol..

[9] Zhang R.J., Zheng J.J., Yu S. (2013). Responses of piles subjected to excavation-induced vertical soil movement considering unloading effect interfacial slip characteristics. Tunn. Undergr. Space Technol..

[10] Zhang Z., Huang M., Xu C., Jiang Y., Wang W. (2018). Simplified solution for tunnel-soil-pile interaction in Pasternak’s foundation model. Tunn. Undergr. Space Technol..

[11] Wang R., Yan B., Wang Y., Li K.J. (2024). Prediction of adjacent single pile deformation induced by tunnel excavation based on the Pasternak model. Tunn. Undergr. Space Technol..

[12] Basile F. (2014). Effects of tunnelling on pile foundations. Soils Found..

[13] Surjadinata J., Hull T.S., Carter J.P., Poulos H.G. (2006). Combined finite-and boundary-element analysis of the effects of tunneling on single piles. Int. J. Geomech..

[14] Franza A., Marshall A.M., Haji T., Abdelatif A.O., Haines T.S., Madabhushi S.P.G. (2017). A simplified elastic analysis of tunnel-piled structure interaction. Tunn. Undergr. Space Technol..

[15] Attewell P.B., Yeates J., Selby A.R. (1986). Soil Movements Induced by Tunneling Their Effects on Pipelines Structures.

[16] Mair R.J. (1993). Unwin memorial lecture 1992. Developments in geotechnical engineering research: Application to tunnels deep excavations. Civ. Eng..

[17] Lee R.G., Turner A.J., Whitworth L.J. (1994). Deformations caused by tunneling beneath a piled structure. International Conference on Soil Mechanics Foundation Engineering.

[18] Forth R.A., Thorley C.B.B. (1996). Hong Kong Islline-predictions performance. Geotechnical Aspects of Underground Construction in Soft Ground.

[19] Boonyarak T., Ng C.W.W. (2012). Tunneling effects on pile group response in Bangkok. GeoCongress 2012: State of the Art Practice in Geotechnical Engineering.

[20] Liu C., Zhang Z., Regueiro R.A. (2014). Pile pile group response to tunneling using a large diameter slurry shield—Case study in Shanghai. Comput. Geotech..

[21] Boonyarak T., Phisitkul K., Ng C.W.W., Teparaksa W., Aye Z.Z. (2014). Observed ground pile group responses due to tunneling in Bangkok stiff clay. Can. Geotech. J..

[22] Mohamad W., Bourgeois E., Le Kouby A., Szymkiewicz F., Michalski A., Branque D., Berthoz N., Soyez L., Kreziak C. (2022). Full scale study of pile response to EPBS tunnelling on a GrParis Express site. Tunn. Undergr. Space Technol..

[23] Khoo C., Mohamad H., Beddelee A.A.A.M., Thansirichaisree P., Ghazali M.F., Nasir M.Y.M. (2025). Effects of advancing tunnel on a loaded pile: Numerical analysis field measurements. J. Rock Mech. Geotech. Eng..

[24] Ma S.J., Hong K.R., Ding Y.G., Guo Y.C., Zhang Y.K. (2025). Effect of shield tunnel cutting pile on composite foundation in Zhengzhou silt: Field test numerical analysis. Results Eng..

[25] Kitiyodom P., Matsumoto T., Kawaguchi K. (2005). A simplified analysis method for piled raft foundations subjected to ground movements induced by tunnelling. Int. J. Numer. Anal. Methods Geomech..

[26] Ding Z., He S.H., Sun Y., Xia T.D., Zhang Q.F. (2021). Comparative study on cyclic behavior of marine calcareous sand and terrigenous siliceous sand for transportation infrastructure applications. Constr. Build. Mater..

[27] Yu J., Sun M., He S., Huang X., Wu X., Liu L. (2021). Accumulative deformation characteristics microstructure of saturated soft clay under cross-river subway loading. Materials.

[28] Jin Z., Liu J., Sun H., Sun M., Xu X. (2024). Influence of gradation range on strong contact network in granular materials. Granul. Matter.

[29] Wei G., Tong J., Liang L., Yu C., Feng G., Wei X. (2026). Evolution law of contact force chain network structure of geotechnical granular materials under unloading stress paths. Materials.

[30] Fuglsang L.D., Ovesen N.K., Craig W.H., James R.G., Schofield A.N. (1988). The Theory of Modelling to Centrifuge Studies, Centrifuge in Soil Mechanics.

[31] Kutter B.L. (1994). Collapse of cavities in sparticle size effects. Proc. Centrifuge.

[32] Marshall A.M. (2009). Tunnelling in Sits Effect on Pipelines Piles. Ph.D. Thesis.

[33] Ghahremannejad B., Surjadinata J., Poon B., Carter J.P. Effects of tunnelling on settlements structural forces within model pile foundations. Proceedings of the International Conference on Physical Modelling in Geotechnics.

[34] Jacobsz S.W., Standing J.R., Mair R.J., Hagiwara T., Sugiyama T. (2004). Centrifuge modelling of tunneling near driven piles. Soils Found..

[35] Lee C.J., Chiang K.H. (2007). Responses of single piles to tunneling-induced soil movements in sandy ground. Can. Geotech. J..

[36] Marshall A.M., Mair R.J. (2011). Tunneling beneath driven or jacked end-bearing piles in sand. Can. Geotech. J..

[37] Boonsiri I., Takemura J. (2015). A centrifuge model study on pile group response to adjacent tunneling in sand. J. JSCE.

[38] Franza A., Marshall A.M. (2017). Centrifuge modelling of tunneling beneath axially loaded displacement non-displacement piles in sand. Geotech. Front..

[39] (2025). Standard Test Methods for Maximum Index Density and Unit Weight of Soils Using a Vibratory Table.

[40] (2025). Standard Test Methods for Minimum Index Density and Unit Weight of Soils and Calculation of Relative Density.

[41] Meguid M.A., Mattar J. (2009). Investigation of tunnel-soil-pile interaction in cohesive soils. J. Geotech. Geoenviron. Eng..

[42] Loganathan N., Poulos H.G., Stewart D.P. (2000). Centrifuge model testing of tunneling-induced ground pile deformations. Géotechnique.

[43] Williamson M.G., Elshafie M.Z.E.B., Mair R.J., Devriendt M.D. (2017). Open-face tunneling effects on nondisplacement piles in clay-part 1: Centrifuge modelling techniques. Géotechnique.

[44] Williamson M.G., Mair R.J., Devriendt M.D., Elshafie M.Z.E.B. (2017). Open-face tunneling effects on non-displacement piles in clay-part 2: Tunneling beneath loaded piles analytical modelling. Géotechnique.

[45] Ong C.W., Leung C.F., Yong K.Y., Chow Y.K. (2005). Centrifuge modelling of pile responses due to tunneling in clay. Proceedings of the Underground Singapore.

[46] Ong C.W., Leung C.F., Yong K.Y., Chow Y.K. (2006). Pile responses due to tunneling in clay. Physical Modelling in Geotechnics, 6th International Conference on Physical Modelling in Geotechnics.

[47] Hartono E., Leung C.F., Shen R.F., Chow Y.K., Ng Y.S., Tan H.T., Hua C.J. (2014). Behaviour of pile above tunnel in clay. Proceedings of the 8th International Conference on Physical Modelling in Geotechnics.

[48] McNamara A.M., Taylor R.N., Stallebrass S.E., Romana M.C. (2003). Influence of tunneling on the behaviour of existing piled foundations. Proceedings of the BGA International Conference on Foundations: Innovations, Obser-Vations, Design Practice.

[49] Ma S.K., Wong K.S., Lv H., Ng C.W.W., Zhao N.F. (2013). Study of effects of tunnel construction on pile group in expansive soil. Rock Soil Mech..

[50] Ma S.K., Shao Y., Lv H., Wong K.S., Ng C.W.W., Chen X., Jiang J. (2016). A study of the long-term influence of twin tunneling on the existing pile group under cyclic variation of groundwater level. Rock Soil Mech..

[51] Gu W.B. (2024). Study on the Mechanism of Tunnel Construction Influences on Lateral Bearing Performance of Adjacent Pile Foundations in Soft Soil Micro Disturbance Control Technology. Ph.D. Thesis.

[52] Lee Y.J., Yoo C.S. Behaviour of a bored tunnel adjacent to a line of loaded piles. Proceedings of the ITA-AITES 2006 World Tunnel Congress 32nd ITA General Assembly: Safety in the Underground Space.

[53] Lee Y., Yoo C. (2008). Two distinctive shear strain modes for pile-soil-tunneling interaction in a granular mass. Proceedings of the 6th International Symposium on Geotechnical Aspects of Underground Construction in Soft Ground.

[54] Broere W., Dijkstra J. (2008). Investigating the influence of tunnel volume loss on piles using photoelastic technique. Proceedings of the 6th International Symposium on Geotechnical Aspects of Underground Construction in Soft Ground.

[55] Zhang W., Gu X., Zhong W., Ma Z., Ding X. (2022). Review of transparent soil model testing technique for underground construction, ground visualization result digitalization. Undergr. Space.

[56] Zhou X.W. (2020). Model Test Numerical Simulation Study of Transparent Soil on Influence of Tunnel Excavation Soil Loss Rate on Pile Foundation of Adjacent Buildings. Master’s Thesis.

[57] Zhu Y., Zeng B., Ye S., He L., Zheng Y., Ma R. (2023). Physical model tests discrete-element simulation of pile soil displacement response induced by DOT shield tunneling based on transparent soil technology. Int. J. Geomech..

[58] Morton J.D., King K.H. (1979). Effects of tunneling on the bearing capacity settlement of piled foundations. Proceedings of the 2nd International Symposium of the Institution of Mining Metallurgy: Tunneling 79.

[59] He S., Lai J., Li Y., Wang K., Wang L., Zhang W. (2022). Pile group response induced by adjacent shield tunnelling in clay: Scale model test numerical simulation. Tunn. Undergr. Space Technol..

[60] Guo C., Yue H., Tao Y., Liu G., Kong F., Lu D., Du X. (2025). Mechanical response of pile group stratum induced by shallow tunneling based on the model test. Tunn. Undergr. Space Technol..

[61] Lee Y.J., Bassett R.H. (2007). Influence zones for 2D pile-soil-tunneling interaction based on model test numerical analysis. Tunn. Undergr. Space Technol..

[62] Shahin H.M., Nakahara E., Nagata M. (2009). Behaviors of ground existing structures due to circular tunneling. Proceedings of the 17th International Conference on Soil Mechanics Geotechnical Engineering.

[63] Shahin H.M., Nakai T., Ishii K., Iwata T., Kuroi S. (2016). Investigation of influence of tunneling on existing building tunnel: Model tests numerical simulations. Acta Geotech..

[64] Liu S.W., Zhang Q.Q., Feng R.F. (2026). Small-scale test on the response of adjacent piles caused by shield tunnel excavation in sand. Transp. Geotech..

[65] Herdarden H.J.A.M., Van der Poel J.T., Van der Schrier J.S. (1996). Ground movements due to tunneling: Influence on pile foundations. Geotech. Asp. Undergr. Constr. Soft Ground.

[66] Bezuijen A., Van der Schrier J.S. (2006). The influence of a bored tunnel on pile foundations. Tunnelling. A Decade of Progress. GeoDelft 1995–2005.

[67] Ran X. (2004). Tunnel Pile Interaction in Clay. Master’s Thesis.

[68] Wu T., Gao Y., Huang C., Zhou Y., Li J. (2024). Mechanical behavior of single group piles with a low cap adjacent to shield tunneling in composite ground: Insights from centrifugal model testing. Geotech. Geol. Eng..

[69] Su J., Pan Y., Niu X., Zhang C. (2025). Effect of shield tunneling on adjacent pile foundations in water-rich strata. Transp. Geotech..

[70] Song G., Marshall A.M. (2020). Centrifuge modelling of tunneling induced ground displacements: Pressure displacement control tunnels. Tunn. Undergr. Space Technol..

[71] Song G., Marshall A.M. (2020). Centrifuge study on the influence of tunnel excavation on piles in sand. J. Geotech. Geoenviron. Eng..

[72] Dias T.G.S., Bezuijen A. (2015). Data analysis of pile tunnel interaction. J. Geotech. Geoenviron. Eng..

[73] Kaalberg F., Teunissen E.H., Van Tol A., Bosch J. (2005). Dutch research on the impact of shield tunnelling on pile foundations. Geotechnical Aspects of Underground Construction in Soft Ground. Proceedings of the 5th International Conference of TC 28 of the ISSMGE, Amsterdam, The Netherlands, 15–17 June 2005.

[74] Selemetas D. (2006). Response of Full-Scale Piles Piled Structures to Tunnelling. Ph.D. Thesis.

[75] Feng S.H., Leung C.F., Chow Y.K., Dasari G.R. Centrifuge modeling of pile responses due to tunneling. Proceedings of the 15th KKCNN Symposium on Civil Engineering.

[76] Fattah M.Y., Hamoud A.A., Al-Tameemi N.T.K. (2015). Deflection of piles due to adjacent tunnelling. Proc. Inst. Civ. Eng.-Geotech. Eng..

[77] Ntritsos N., Anastasopoulos I. (2020). Tunnelling-induced settlement of pile groups: Effects of pile group stiffness and size. Tunn. Undergr. Space Technol..

[78] Franza A., Marshall A.M. (2019). Centrifuge modeling of pile responses to tunneling in sand. J. Geotech. Geoenviron. Eng..

[79] Lee C.J., Kao C.M., Chiang K.H. (2003). Pile response due to nearby tunneling. Proceedings of the BGA International Conference on Foundations: Innovations, Observations, Design Practice.

[80] Haji T., Marshall A.M., Franza A. (2018). Centrifuge testing of a piled structure affected by tunneling. Tunn. Undergr. Space Technol..

[81] Sun M., Yu J., He S., Ding Z. (2021). Cyclic degradation characteristics of undisturbed soft clay considering anisotropy. Adv. Mater. Sci. Eng..

[82] He S.H., Goudarzy M., Ding Z., Sun Y., Xu T., Zhang Q.F. (2022). Small-strain shear modulus (G max) and microscopic pore structure of calcareous sand with different grain size distributions. Granul. Matter.

[83] Ding Z., Chen Y., He S.H., Sun M.M. (2023). Investigating the long-term deformation behaviour and non-coaxiality of siliceous sand under traffic cyclic loading. Constr. Build. Mater..

[84] Zhang Y.Z., Li X.Z., Yu G.H. (2016). Chloride transport in undersea concrete tunnel. Adv. Mater. Sci. Eng..

[85] Liu J.Y., Wautier A., Zhou W., Nicot F., Darve F. (2022). Incremental shear strain chain: A mesoscale concept for slip lines in 2D granular materials. Granul. Matter.

[86] Xu C.J., Lin Z.R., Jiang Y.L., Shi Y.F., Fan X.Z., Xiong Z., Liu Y.F. (2022). Research on the spatial effect of foundation pit under asymmetric loads. Front. Mater..

[87] Zhang Y.Z., Gu L.Y., Zhang Q.L. (2022). Durability of manufactured sand concrete in atmospheric acidification environment. Case Stud. Constr. Mater..

[88] Zhang Y.Z., Zhang X.M., Jin F., Zhao X.Y. (2024). Impact of stone powder content on corrosion resistance in reinforced concrete under stray current and chloride interactions. Materials.

[89] Zhang L.S., Li Y., Wei X., Liang X., Zhang J.H., Li X.C. (2024). Unconfined compressive strength of cement-stabilized Qiantang river silty clay. Materials.

[90] Wang C.Q., Xie J., Shen Y.G., Jiang J.Q. (2022). Research on the mechanical behavior of a steel–concrete composite link slab on a simply supported girder bridge. Metals.

[91] Wang C.Q., Yin C.L., Zou Y., Ping B.Y., Wu X., Liao J., Sun M.M. (2023). Numerical investigations on seismic behavior of segmental assembly of concrete filled steel tube piers with external replaceable energy-dissipating links. Materials.

